# Supplementary material for: A network-based approach reveals the dysregulated transcriptional regulation in non-alcoholic fatty liver disease
Source: iScience. 2021 Oct 5;24(11):103222. doi: 10.1016/j.isci.2021.103222 (PMC8529555; doi:10.1016/j.isci.2021.103222)
Supplement: Document S1. Figures S1–S8 [file mmc1.pdf]

iScience, Volume *24*

Supplemental information

A network-based approach reveals the dysregulated transcriptional regulation in non-alcoholic fatty liver disease

Hong Yang, Muhammad Arif, Meng Yuan, Xiangyu Li, Koeun Shong, Hasan Türkez, Jens Nielsen, Mathias Uhlén, Jan Borén, Cheng Zhang, and Adil Mardinoglu

Supplementary Appendix

This PDF file includes:

Supplementary Figures. S1 - S8

SUPPLEMENTARY FIGURE LEGEND

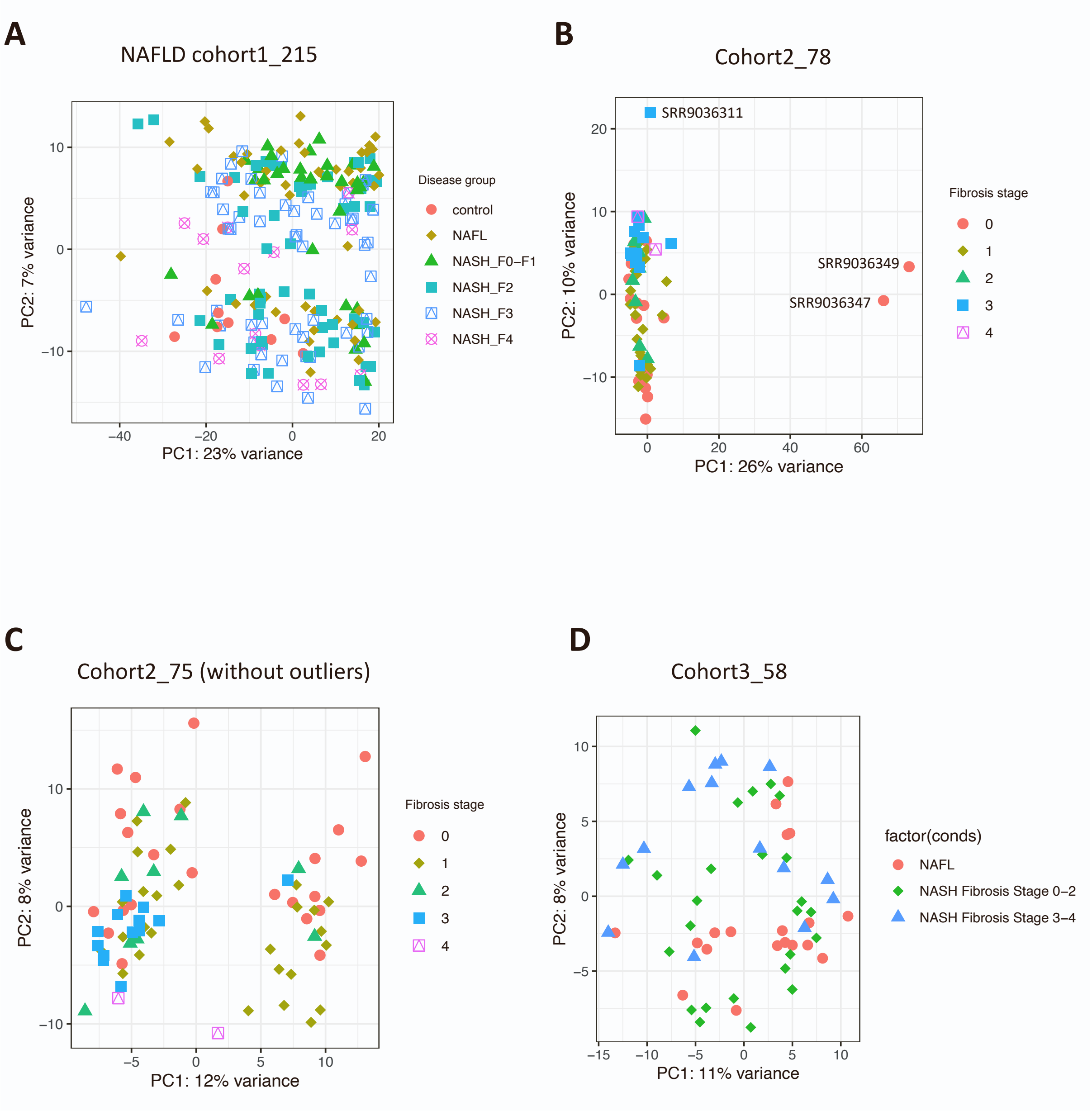

Figure S1. PCA analysis for RNA-seq datasets. Related to STAR Methods.

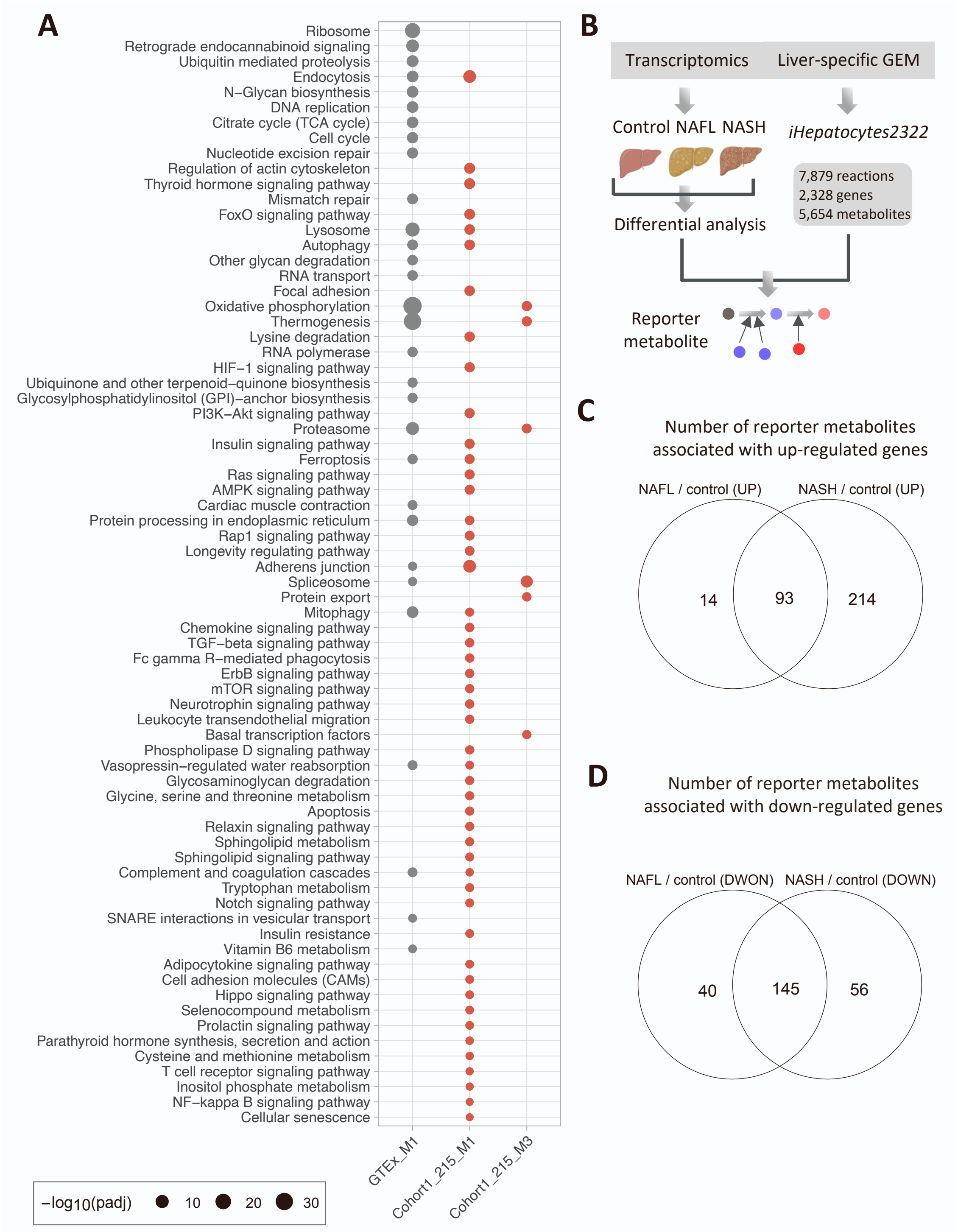

Figure S2. Functional enrichment analysis of perturbated gene modules in NAFLD and reporter metabolites associated with NAFLD. (**A**) Dot-plot heatmaps showing KEGG pathways enriched in different modules. Color differences of dots indicate the studied cohort (GTEx or NAFLD) in which the module detected. The size of each dot is proportional to the significance (-$\log_{10}$(padj). Related to Figure 1E. (**B**) Reporter metabolites analysis was used for the analysis of transcriptomics data from the NAFLD cohort. (**C**) The Venn diagram shows the number of reporter metabolites associated with up-regulated genes in either NAFL vs control or NASH vs control. (**D**) The Venn diagram shows numbers of reporter metabolites associated with down-regulated genes in either NAFL vs control or NASH vs control. Related to Figure 2.

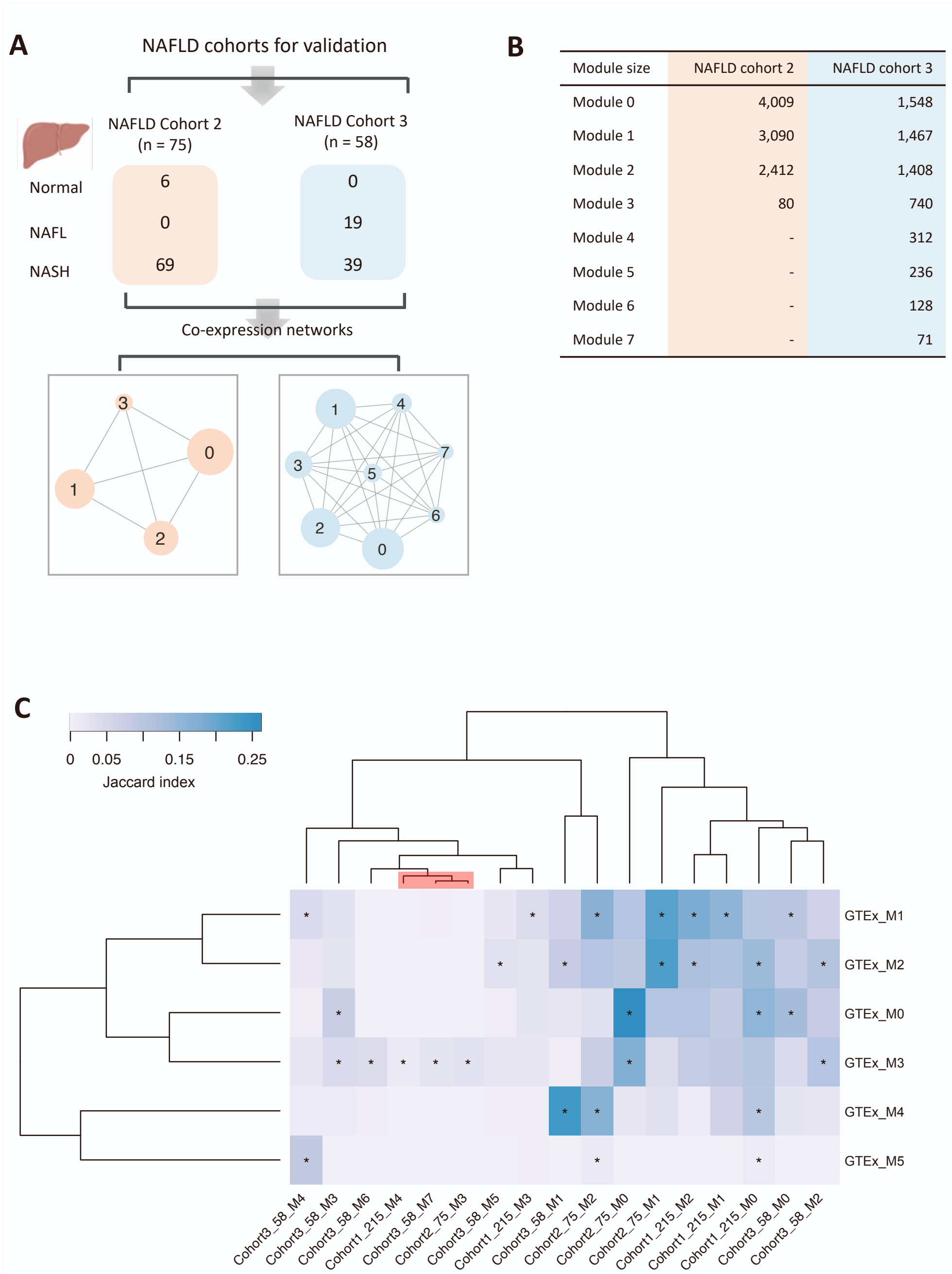

Figure S3. Validation of disease-associated perturbated modules by two independent NAFLD cohorts. (**A**) Sample information of NAFLD cohort 2 and 3 for validation. Transcriptome data of liver tissue were obtained from NAFLD cohorts with 75 and 58 samples ranging from normal, NAFL, NASH, respectively. Spearman rank-order correlation coefficient analysis was applied to calculate the correlation between gene pairs after removing the lowly expressed genes (TPM <1), and the Leiden algorithm was used to detect modules of significantly correlated genes. The label (number) of the module assigned by the algorithm. (**B**) The numbers of genes consist of the individual module in each cohort. (**C**) Hierarchical clustering of Jaccard Index between module pairs from GTEx cohort and all three NAFLD cohorts. Colour scales representing the range of the Jaccard index. Asterisk indicates the statistical significance of the overlap between gene members in any two modules from the different cohort. The test was performed by a hypergeometric test. The overlap was evaluated as significant when the p-value < 0.05. NAFLD, non-alcoholic fatty liver disease; NAFL, non-alcoholic fatty liver; NASH, non-alcoholic steatohepatitis; TPM, transcripts per kilobase million. Related to Figure 3

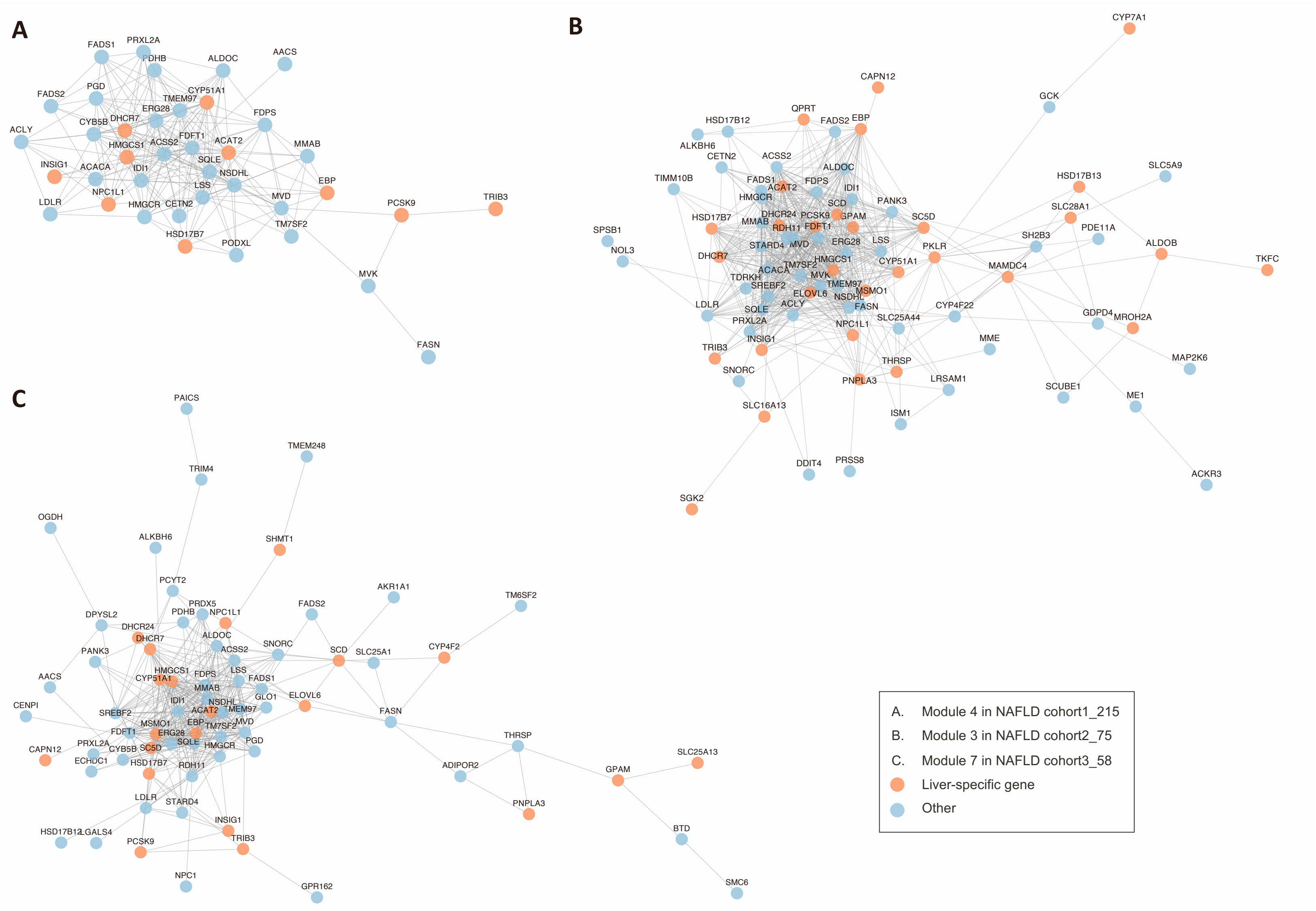

Figure S4. Visualization of disease-associated modules constructed by Cytoscape with "Preferred Layout". (**A**) cohort1_215_M4. (**B**) cohort2_75_M3. (**C**) cohort3_58_M7. Orange colour nodes are liver-specific genes based on HPA. Related to Figure 4C-E.

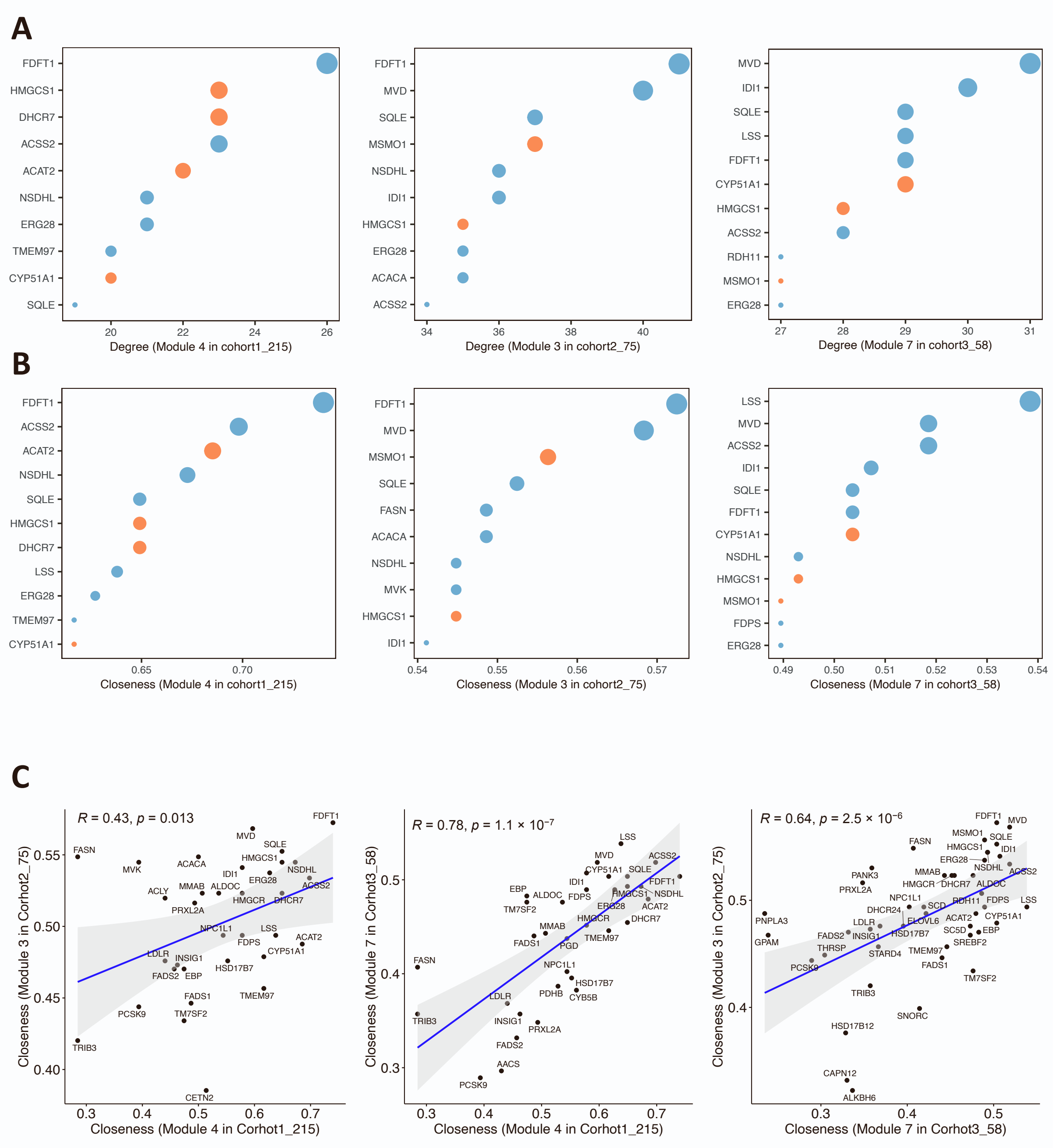

Figure S5. Topological properties of gene in disease-associated modules. (**A, B**) Top 10 genes with highest degree and closeness in disease-associated modules. (**C**) Correlation between closeness among disease-associated modules from different cohorts. The correlation was evaluated by Spearman correlation coefficients. Related to Figure 4C-E.

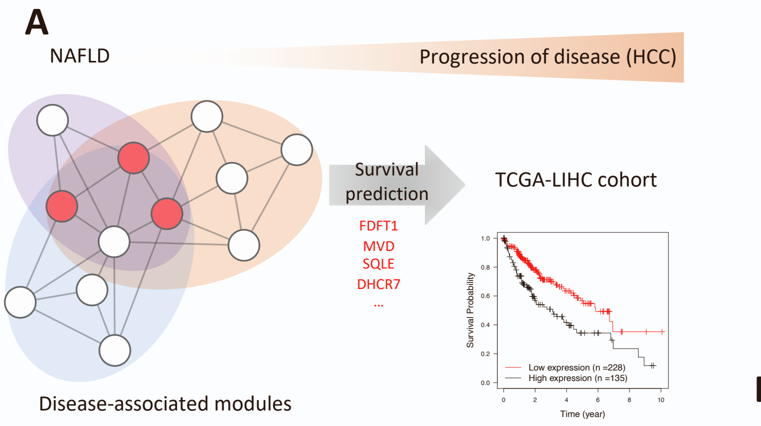

B

Module	Size	KM (Available)	Significant association	favourable survival of patients	unfavourable survival of patients
Cohort1_215_M4	38	37	19	0	19
Cohort2_75_M3	80	77	39	11	28
Cohort3_58_M7	71	71	40	9	31

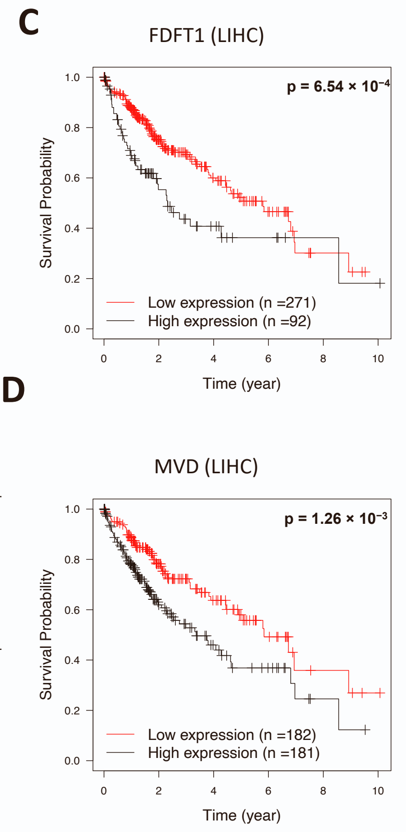

Figure S6. Validation of topological features in an HCC cohort. (**A**) Schematic framework of survival analysis for the genes in disease-associated modules using the liver cancer dataset in TCGA database. (**B**) Summary of Kaplan–Meier analysis for each of the module. (**C, D**) The Kaplan–Meier plots for FDFT1 and MVD with high-connectivity in cohort1_215_M4 and cohort2_75_M3 and cohort3_58_M7, respectively. Related to Figure C-E.

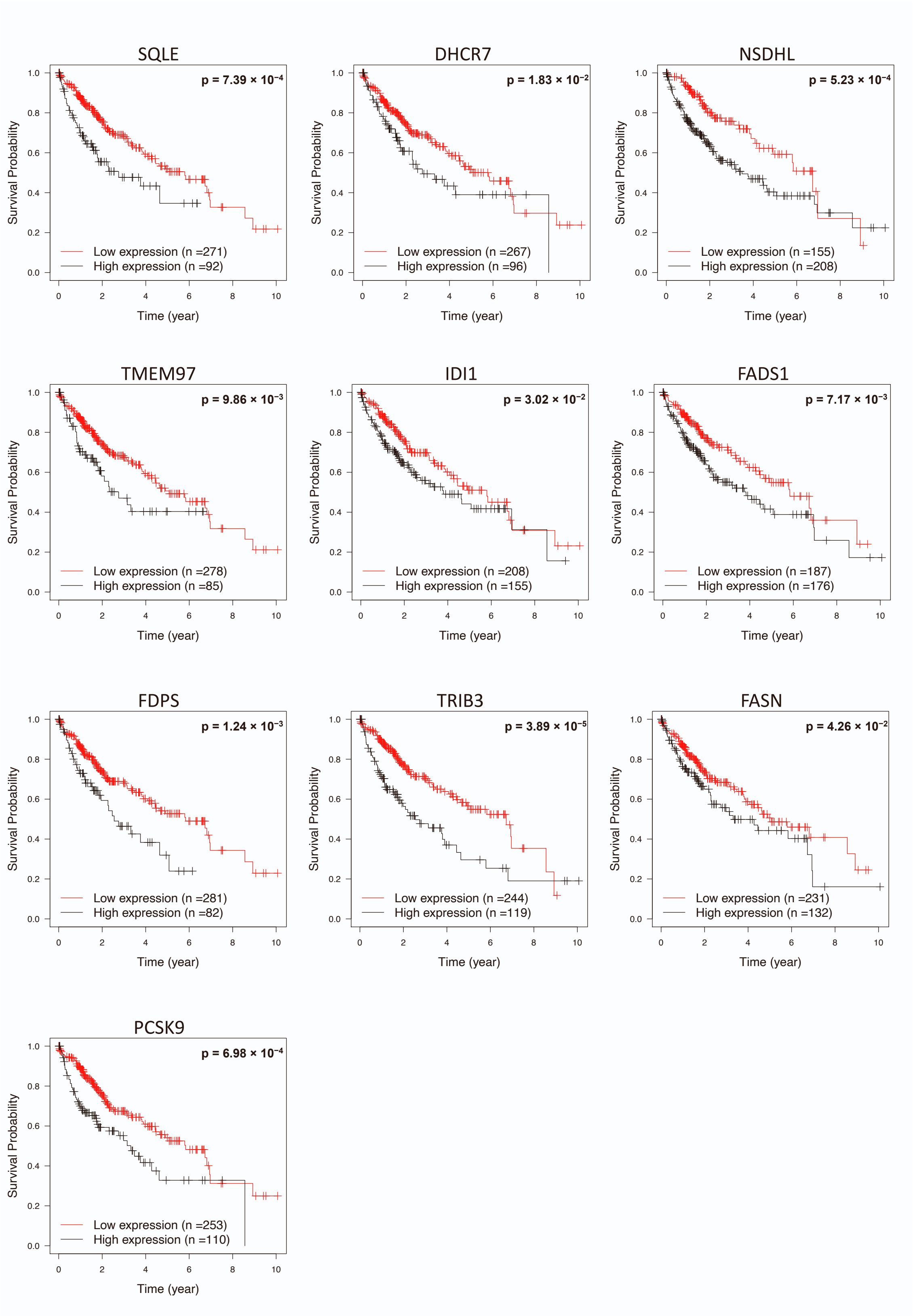

Figure S7. The Kaplan-Meier plots showing the high expression of genes shared by disease-associated modules (including SQLE, DHCR7, NSDHL, TMEM97, IDI1, FADS1, FDPS, TRIB3, FASN, and PCSK9) are significantly associated with poor outcome of patients. For each plot, the log-rank test was performed to compare survival curves between high-expression group and low-expression group. Related to Figure C-E.

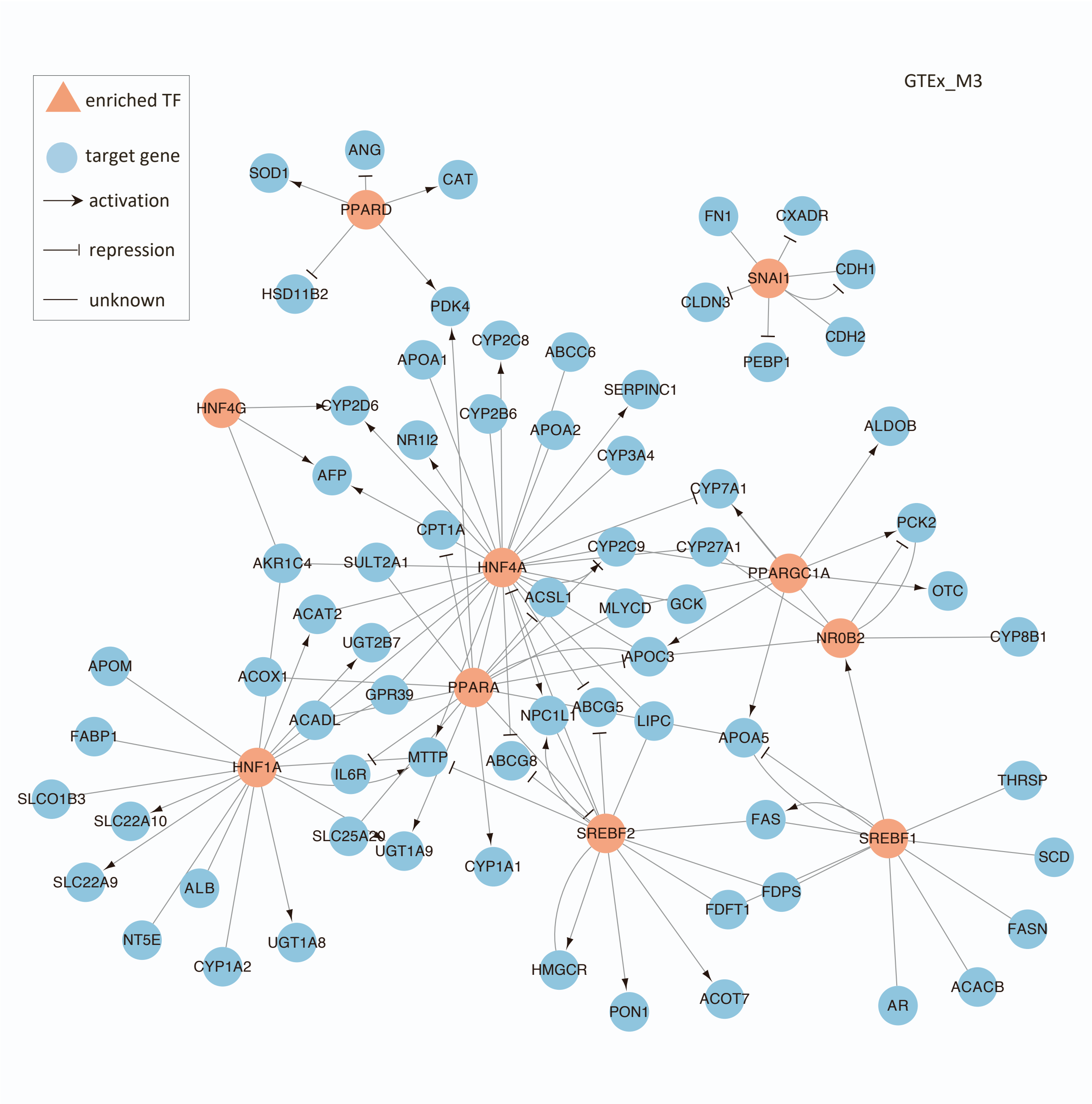

Figure S8. Regulatory relationship between an enriched transcription factor and associated target genes in GTEx_M3. Related to Figure 5.
